# Comprehensive Evaluation of Peonidin: Antioxidant and Multi-Enzyme Inhibitory Abilities with Molecular Docking Insights

**DOI:** 10.3390/molecules31162931

**Published:** 2026-08-21

**Authors:** Hasan Karageçili, Emrah Yerlikaya, Adem Ertürk, Kübra Aslan, Hülya Akıncıoglu, İlhami Gülçin

**Affiliations:** 1 Nursing Department, Faculty of Health Sciences, Siirt University, 56100 Siirt, Türkiye; 2 Nutrition and Dietetics Department, Faculty of Health Sciences, Siirt University, 56100 Siirt, Türkiye; emrahyerlikaya@siirt.edu.tr; 3 Department of Chemistry, Faculty of Science, Atatürk University, 25240 Erzurum, Türkiye; a.erturk@atauni.edu.tr (A.E.); kubra.aslan@atauni.edu.tr (K.A.); 4 Faculty of Pharmacy, Department of Biochemistry, Agri Ibrahim Cecen University, 04100 Agri, Türkiye; hgakincioglu@agri.edu.tr; 5 Rectorate of Agri Ibrahim Cecen University, 04100 Agri, Türkiye

**Keywords:** peonidin, antioxidant, α-glycosidase, antimicrobial, acetylcholinesterase, carbonic anhydrase, flavonoid, molecular docking

## Abstract

Anthocyanins are water-soluble plant pigments. They give many plants, fruits, vegetables, and cereal kernels their red, purple, and blue colors. This research aims to reveal the biological properties of peonidin as an anthocyanin. To comprehend the antioxidant capabilities of peonidin, 2,2′-azino-bis(3-ethylbenzothiazoline-6-sulfonic acid) radical (ABTS^•+^), N,N-dimethyl-p-phenylenediamine dihydrochloride radical (DMPD^•+^), and 1,1-diphenyl-2-picrylhydrazyl free radical (DPPH^•^) scavenging, Fe^3+^-2,4,6-tris(2-pyridyl)-s-triazine (TPTZ), and Cu^2+^ reducing assays were recorded. The IC_50_ values for peonidin against ABTS^•+^, DMPD^•+^ and DPPH^•^ scavenging capabilities were determined to compare with standard antioxidants. ABTS^•+^ radical scavenging activity of peonidin had an IC_50_ value of 15.40 μg/mL, while the IC_50_ values for BHA, BHT, Trolox, and α-Tocopherol were 12.82, 11.78, 12.67, and 10.83 μg/mL, respectively. DPPH radical scavenging activity of peonidin had an IC_50_ value of 41.63 μg/mL, while the IC_50_ values for BHA, BHT, Trolox, and α-Tocopherol were 8.45, 23.10, 6.30, and 18.73 μg/mL, respectively. Enzyme inhibition was studied to investigate the effects of peonidin. The K_i_ values of peonidin were 114.33, 63.02, 2.99, 9.76, and 15.14 nM toward hCA I, hCA II, AChE, BChE, and α-glycosidase enzymes, respectively. Furthermore, peonidin’s interactions with target enzymes BChE, hCA I, hCA II, AChE, and α-glycosidase were investigated by molecular docking. The results suggest that antioxidant-rich peonidin is a plant-based compound with potential use in the treatment of glaucoma, Alzheimer’s disease, and diabetes.

## 1. Introduction

Plants have long been utilized as natural remedies to cure a variety of illnesses. As a result, plants are generally recognized as significant reservoirs of bioactive phytochemicals for the discovery of new medicines, and substantial research is ongoing globally. Furthermore, about 80% of the 122 plant-derived medicines now in clinical trials were derived from plant species with a well-documented history of traditional medicinal usage [1]. Herbal treatments are extensively recognized for their long-standing use in treatment regimens and are frequently chosen to treat multiple conditions because of their reduced adverse effects and active substances. Conversely, synthetic medications typically have more adverse effects, even when they could be more successful at treating a single ailment. Herbal treatments’ delayed onset of effect is their main drawback, which requires long-term use to achieve optimal results [2].

Anthocyanins are water-soluble plant pigments with biological activity, often referred to as “cell sap pigments”. They give plants their vibrant colors. To date, more than 700 anthocyanins have been identified, isolated from natural plant sources such as fruits, vegetables, and grains, with the number continuing to grow [3]. Anthocyanins constitute a subclass of naturally occurring flavonoids that include several major pigments, namely cyanidin, peonidin, delphinidin, malvidin, pelargonidin, and petunidin. Recently, the activities of anthocyanins have come to the forefront, ranging from their roles in disease prevention to their active roles in anti-inflammatory effects, diabetes, cancer, neurological disorders, liver and kidney protection, and immunomodulation [4]. Anthocyanins are a type of flavonoid with numerous biological activities, including anti-tumor, antiviral, antioxidant, anti-atherosclerotic, anti-diabetic, and tyrosinase-inhibiting effects. Cyanidin-3-galactoside, cyanidin, and peonidin are the three most common active anthocyanins, all of which possess antioxidant and anti-diabetic effects [5]. Derivatives of peonidin and quercetin contribute significantly to the antioxidant capacity of fruits, the inhibition of enzymes that hydrolyze carbohydrates, and antiproliferative effects [6]. Peonidin 3-O-glucoside significantly reduced intracellular lipid accumulation in an in vitro model of non-alcoholic fatty liver disease, and it has been shown to prevent oxidative stress by preventing the overproduction of superoxide anions and reactive oxygen species, raising glutathione levels, and boosting the activity of enzymes such as superoxide dismutase, glutathione peroxidase, and catalase [7]. Although anthocyanins are widely known for their variety of biological activities, it remains a significant scientific problem to pinpoint the precise anthocyanin that is principally responsible for antioxidant activity. Understanding this connection might help create peonidin-based functional foods and treatment strategies with potential health advantages as well as offer insightful information on the antioxidant capacity of peonidin.

Reactive oxygen species (ROS) are created in excessive amounts within metabolic processes and may result in oxidative stress. They can oxidize and destroy essential elements of cell membranes, including lipids, proteins, and nucleic acids. ROS, which induce cellular death and alter the functioning of genetic mechanisms, are associated with the progression of many diseases in humans [8]. However, antioxidants can prevent or lessen oxidative stress and may delay or alleviate illnesses caused by oxidative damage. Antioxidants that are both enzymatic and non-enzymatic are the main classes of antioxidants [9]. Antioxidants, which are not synthesized by the body and can be obtained through foods rich in vitamins, minerals, and bioactive compounds, can help strengthen the body’s antioxidant system [10]. Free radicals like peroxides, hydroperoxides, and lipid peroxyls are scavenged by antioxidants such as flavonoids, phenolic acids, tannins, and phenolic diterpenes, which also block the oxidation process that causes progressive illnesses [11]. 

Protection against chronic conditions including cancer, Parkinson’s disease (PD), cataracts, type-2 diabetes mellitus (T2DM), cardiovascular diseases, and Alzheimer’s disease (AD) may be achieved through the use of antioxidants [12]. Cholinergic enzymes, acetylcholinesterase (AChE) and butyrylcholinesterase (BChE), share certain structural similarities, including the presence of a catalytic center. Research on novel inhibitors of cholinesterase seems to be a key approach to bringing different drug options to market for the care of Alzheimer’s disease and associated variants of dementia [13]. Being the fourth most common cause of mortality in advanced nations, T2DM is regarded as an epidemic. The etiology of T2DM, which encompasses lifestyle choices, dyslipidemia, and genetic predisposition, is a complex condition due to the multitude of factors involved. Inhibiting α-glycosidase, one of the primary digestive enzymes responsible for accelerating the digestion of polysaccharides in the diet, is the primary aim of T2DM treatment [14]. 

Carbonic anhydrase enzymes (CAs) perform various metabolic and biochemical functions, such as urea production, gluconeogenesis, and fat synthesis. Conditions such as infections, spasms, glaucoma, and cancer may be alleviated by inhibiting CA enzymes [15]. Almost all natural fruits, vegetables, and foods contain roughly the same antioxidants. In this sense, antioxidants found in natural foods, vegetables, plants, and fruits are well-known for their ability to readily neutralize ROS or free radicals. Consequently, reliable, naturally occurring plant-based antioxidants are being sought after and considered as complementary and alternative options to synthetic antioxidants, which have harmful effects [16]. 

Medicinal herbs have been the center of countless research studies and are among the most important sources of natural antioxidants. The phenolic content in medicinal plants is quite high. Phenolic compounds are an important class of plant secondary metabolites that have been reported to exert protective effects against a wide range of chronic degenerative diseases, including cataracts, cardiovascular disease, cancer, hypercholesterolemia, diabetes mellitus, and rheumatoid arthritis [17,18]. Flavonoids are known as antioxidant phenols found in potent plant-based foods that help neutralize free radicals not only in organs but also in tissues and cells. Thanks to the plant compounds found in the diet, the vascular endothelium functions more effectively in reducing the risk of high blood pressure, cardiovascular diseases, T2DM, and AD [19].

Although peonidin is a naturally occurring anthocyanidin with reported biological properties, its antioxidant capacity and inhibitory effects on metabolically relevant enzymes have not yet been comprehensively evaluated using multiple complementary assays. Therefore, the present study aimed to systematically investigate the in vitro antioxidant, enzyme-inhibitory, and antimicrobial properties of peonidin. Its antioxidant potential was assessed using Fe^3+^- and Cu^2+^-reducing assays together with DPPH, ABTS, and DMPD radical-scavenging methods. The inhibitory effects of peonidin were evaluated against human carbonic anhydrase I and II (hCA I and hCA II), acetylcholinesterase (AChE), butyrylcholinesterase (BChE), and α-glycosidase, which are relevant biochemical targets in disorders associated with oxidative stress, cholinergic dysfunction, and glucose metabolism. Its antimicrobial activity was also examined against selected Gram-positive and Gram-negative bacterial strains. In addition, molecular docking analyses were conducted to characterize the possible binding modes and interactions of peonidin within the active sites of the target enzymes. By integrating experimental and computational approaches, this study aimed to provide a more comprehensive biochemical profile of peonidin while identifying areas requiring further mechanism and in vivo investigation.

## 2. Results

### 2.1. Antioxidant Studies

**Figure 1:**
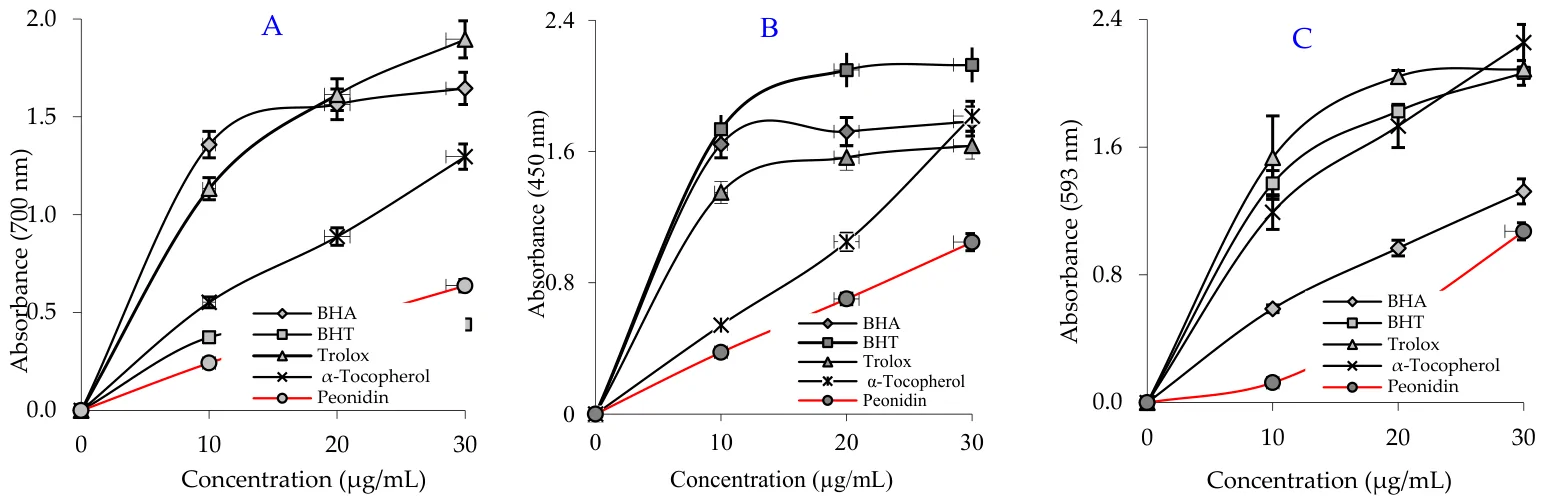
(**A**) Ferric ions (Fe^3+^) reduction capacity of peonidin and the standards, (**B**) Cupric ions (Cu^2+^) reduction capacity of peonidin and the standards, (**C**) Fe^3+^-TPTZ reduction capacity of peonidin and the standards; at a concentration of 30 μg/mL.

The antioxidant potential of natural compounds is being investigated using various simple, cost-effective in vitro antioxidant methods. Antioxidant compounds are generally studied using methods such as the reduction of potassium ferricyanide and copper ions (Cu^2+^), as well as the scavenging of DMPD^•+^, ABTS^•+^, and DPPH^•^. In one of the previously conducted studies, a ferric hexacyanoferrate molecule (Fe_4_[Fe(CN)_6_]) with an absorption at 700 nm was formed by adding Fe^3+^ to the reduced product following the addition of peonidin. Fe[(CN)_6_]^3+^ is reduced to Fe[(CN)_6_]^2+^ in the presence of reducing molecules [20]. Furthermore, as indicated in Table 1 and Figure 1A, peonidin demonstrated an acceptable Fe^3+^ reduction capacity. This capacity was observed to be highly significant (*p* < 0.01). The reduction capacity of peonidin, Trolox, α-tocopherol, BHT, and BHA increased in parallel with increasing peonidin concentrations. The Fe^3+^ reduction capacity of peonidin and the reference compounds was determined as follows: Trolox (λ_700_: 1.896 ± 0.141, r^2^: 0.9086) > BHA (λ_700_: 1.646 ± 0.027, r^2^: 0.7418) > α-Tocopherol (λ_700_: 1.297 ± 0.014, r^2^: 0.9896) > Peonidin (λ_700_: 0.637 ± 0.020, r^2^: 0.9970) > BHT (λ_700_: 0.440 ± 0.006, r^2^: 0.7230) at 30 μg/mL.

The results showed that peonidin has an effective Fe^3+^ reduction capacity, but that BHA, α-tocopherol, BHT, and Trolox have lower Fe^3+^ reduction capacity.

The reduction capacities of cupric ions (Cu^2+^) and peonidin at the same amounts (30 μg/mL) are shown in Table 1 and Figure 1B. A positive correlation has been identified among various peonidin quantities and the ability to reduce Cu^2+^. It has been found that a higher amount (10–30 µg/mL) of peonidin is required to reduce Cu^2+^. The Cu^2+^ reduction capacity of peonidin and the reference compounds at 30 µg/mL was established along these lines: BHT (λ_450_: 2.128 ± 0.008, r^2^: 0.7744) > α-Tocopherol (λ_450_: 1.815 ± 0.056, r^2^: 0.9906) > BHA (λ_450_: 1.783 ± 0.045, r^2^: 0.6636) > Trolox (λ_450_: 1.635 ± 0.015, r^2^: 0.7407) > peonidin (λ_450_: 0.701 ± 0.020, r^2^: 0.9992) at a concentration of 30 μg/mL. The findings indicate that peonidin has a strong capacity to reduce Cu^2+^, although it is lower than that of the standard antioxidants BHT, Trolox, BHA, and α-tocopherol.

The reduction capacities of Fe^3+^-TPTZ and peonidin at the same concentration (30 μg/mL) are given in Table 1 and Figure 1C. The Fe^3+^-TPTZ reduction capabilities of peonidin and the reference compounds at 30 μg/mL were determined as follows: α-tocopherol (λ_593_: 2.256 ± 0.112, r^2^: 0.9527) > Trolox (λ_593_: 2.089 ± 0.012, r^2^: 0.8013) > BHT (λ_593_: 2.065 ± 0.077, r^2^: 0.8647) > BHA (λ_593_: 1.322 ± 0.078, r^2^: 0.9842) > peonidin (λ_593_: 1.072 ± 0.020, r^2^: 0.9347) at 30 μg/mL. The results revealed that peonidin’s Fe^3+^-TPTZ reduction capacity was lower than that of the reference antioxidants BHT, Trolox, BHA, and α-tocopherol. Still, they were close to and comparable to their standard activities.

**Figure 2:**
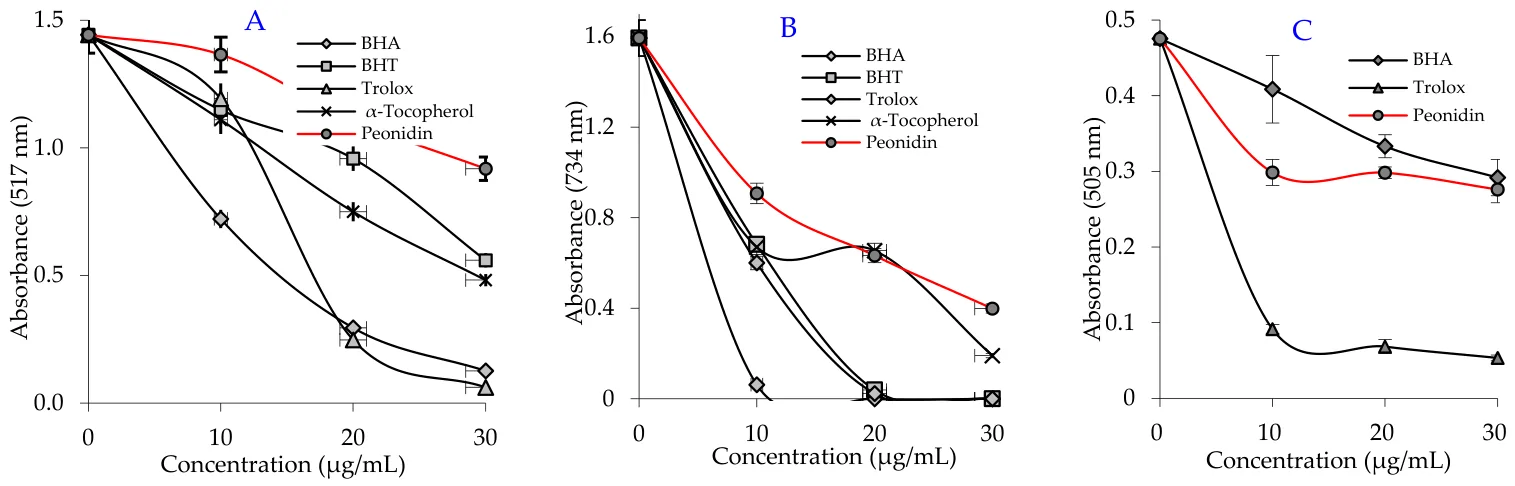
Radical scavenging capacities of peonidin and reference antioxidants: (**A**) DPPH^•^ scavenging, (**B**) ABTS^•+^ scavenging, (**C**) DMPD^•+^ scavenging effects.

The antioxidant qualities of pure or recently synthesized substances are frequently assessed using the radical scavenging test. The best-known and frequently utilized radical scavenging method for this purpose is the DPPH scavenging method. In the DPPH free radical scavenging method, the IC_50_ value for peonidin was determined to be 41.63 μg/mL (r^2^: 0.9642) (Table 2 and Figure 2A). In contrast, the IC_50_ levels were 6.30 μg/mL (r^2^: 0.9239) for Trolox, 8.45 μg/mL (r^2^: 0.9952) for BHA, 18.73 μg/mL (r^2^: 0.9966) for α-tocopherol, and 23.10 μg/mL (r^2^: 0.9818) for BHT. Peonidin exhibited measurable DPPH radical-scavenging activity; however, its higher IC_50_ value compared with those of the reference antioxidants indicates a lower scavenging potency under the applied experimental conditions. The experiments show that the compounds’ capacity to scavenge free radicals is significantly impacted by the hydroxyl groups in their aromatic rings [21]. These findings indicate that peonidin possesses an effective DPPH free radical scavenging capacity in comparison with flavonoid aromatic compounds studied in the literature.

According to the findings, Peonidin exhibited appreciable ABTS radical-scavenging activity; however, its higher IC_50_ value than those of the reference antioxidants indicates slightly lower scavenging potency under the applied experimental conditions. The DPPH^•^, DMPD^•+^ and ABTS^•+^ assays are also frequently utilized to analyze the radical-scavenging capability of concentrated compounds, beverages, extracts, and slurries [22]. In this study, the IC_50_ value of peonidin for the ABTS^•+^ free radical scavenging assay was demonstrated to be 15.40 µg/mL (r^2^: 0.9908) (Table 2 and Figure 2B). Furthermore, the IC_50_ levels were 10.83 μg/mL (r^2^: 0.9304) for α-tocopherol, 11.78 μg/mL (r^2^: 0.9468) for BHT, 12.67 μg/mL (r^2^: 0.9899) for Trolox, and 12.82 μg/mL (r^2^: 0.9966) for BHA. The findings indicate that peonidin, a phenolic compound, possesses effective ABTS^•+^ radical scavenging activity in comparison with the reference compounds used.

The DMPD radical was generated in vitro by oxidation with ferric chloride. An IC_50_ value of 31.53 μg/mL (r^2^: 0.6918) was recorded for peonidin (Table 2 and Figure 2C). In contrast, the IC_50_ values were measured as 10.19 μg/mL (r^2^: 0.6735) for Trolox and 40.77 μg/mL (r^2^: 0.9872) for BHA. The findings demonstrated that Peonidin exhibited measurable DMPD radical-scavenging activity; however, its higher IC_50_ value than those of BHA and Trolox indicates comparatively weaker scavenging potency under the applied experimental conditions.

### 2.2. Enzyme Inhibition Studies

**Figure 3:**
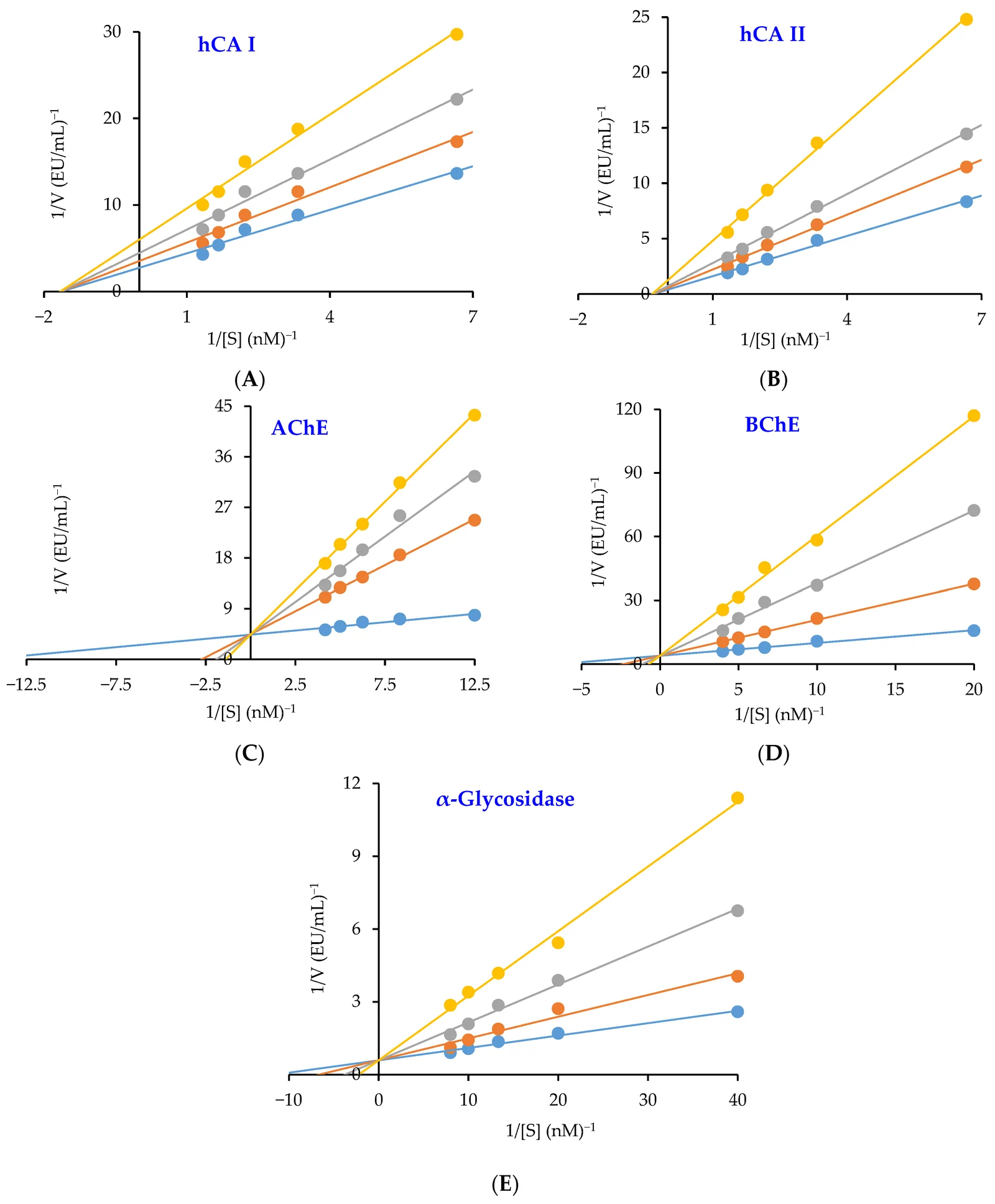
Lineweaver-Burk plots of peonidin towards carbonic anhydrase I (CA I) (**A**), carbonic anhydrase II (CA II) (**B**), acetylcholinesterase (AChE) (**C**), butyrylcholinesterase (BChE) (**D**), and α-glycosidase (**E**).

Active site, thereby inhibiting the activity of CA isoenzymes. According to a study profiling the predominant intracellular hCA I and hCA II isoenzymes, peonidin exhibited impressive Ki values of 114.33 ± 18.72 nM and 63.02 ± 18.56 nM, respectively, relative to AZA inhibitor values (Table 3 and Figure 3A,B). In comparison, AZA, a clinically used CA inhibitor, exhibited Ki values of 83.28 ± 11.84 nM against hCA I and 56.97 ± 2.03 nM against hCA II isoforms. The physiologically predominant cytosolic hCA I and hCA II are found in almost every cell and are linked to several illnesses, including edema, epilepsy, and glaucoma.

The cholinergic enzymes AChE and BChE, which are targeted to reduce AD signs, were effectively inhibited by peonidin with Ki values of 2.99 ± 0.23 and 9.76 ± 3.23 nM, respectively (Table 3, Figure 3C,D). The selectivity index (K_i-AChE_/K_i-BChE_) was determined to be 0.31 for both cholinergic enzymes. Accordingly, the affinities of peonidin for the two cholinergic enzymes differ significantly. Furthermore, tacrine exhibited Ki values of 1.85 ± 0.31 nM for AChE (Figure 3C) and 1.52 ± 0.01 nM for BChE (Figure 3D). AD is the most prevalent form of dementia and primarily affects older adults. AChE is the principal cholinesterase at cholinergic synapses and neuromuscular junctions, where it rapidly hydrolyzes acetylcholine and terminates cholinergic neurotransmission [23].

**Table 3:** Enzyme inhibition values of Peonidin towards carbonic anhydrase isoenzymes I and II (CA I and CA II), acetylcholinesterase (AChE), butyrylcholinesterase (BChE), and α-glycosidase (α-Gly) enzymes.

Compounds	IC_50_ (nM)										Ki (nM)				
	hCA I	r^2^	hCA II	r^2^	AChE	r^2^	BChE	r^2^	α-Gly	r^2^	hCA I	hCA II	AChE	BChE	α-Gly
Peonidin	115.52	0.9900	57.76	0.9879	14.15	0.9875	13.59	0.9800	33.01	0.9860	114.33 ± 18.72	63.02 ± 18.56	2.99 ± 0.23	9.76 ± 3.23	15.14 ± 4.93
AZA *	63.01	0.9794	40.77	0.9849	-	-	-	-	-	-	83.28 ± 11.84	56.97 ± 2.03	-	-	-
TAC **	-	-	-		8.89	0.9849	6.86	0.9800	-	-	-	-	1.85 ± 0.31	1.52 ± 0.01	-
ACR ***	-	-	-		-	-	-		22,800	-	-	-	-	-	-

* Acetazolamide (AZA) is the standard inhibitor for both hCA I and hCA II isoenzymes. ** Tacrine (TAC) is the standard inhibitor for AChE and BChE enzymes. *** Acarbose (ACR) is the standard inhibitor for α-glycosidase as (IC_50:_ 22,800 µM) [24].

The characteristic symptoms of T2DM include hyperglycemia and inadequate intrinsic insulin production or action. High blood sugar levels are linked to this metabolic disease. The current investigation has concentrated on inhibiting α-glycosidase to regulate the breakdown of carbohydrates [25,26]. Peonidin has a Ki value of 15.14 ± 4.93 nM against the α-glycosidase enzyme (Table 3 and Figure 3E). The results unequivocally show that peonidin exhibits effective α-glycosidase inhibition when assessed in comparison with acarbose (IC_50_: 22,800 µM), a typical α-glycosidase inhibitor and an antidiabetic drug for T2DM [24,25].

### 2.3. Antimicrobial Studies

Antimicrobial tests revealed that peonidin exhibited inhibitory effects on *S. aureus* and *E. coli*, whereas it did not indicate any antimicrobial activity towards *P. aeruginosa* and *K. pneumoniae* strains. According to disk diffusion results, an inhibition zone of 13 mm was measured at a peonidin concentration of 40 µg/mL for *S. aureus* and 12 mm at a concentration of 80 µg/mL for *E. coli*.

### 2.4. Molecular Docking Studies

The insertion and binding energy scores of peonidin and reference molecules (CA I, II enzyme inhibitor acetazolamide, AChE, BChE enzymes inhibitor tacrine, and α-glycosidase enzyme inhibitor acarbose) obtained as a result of the study are given in Table 4. Furthermore, the protein’s pocket grid size and the residues and distances (Å) involved in hydrophobic interactions, which are the summary results of the study, are given in Table 5, and 2D and 3D zoomed-in insertion images are given in Table 6.

#### Docking Validation Studies

Redocking validation was performed using the native co-crystallized ligands extracted from each PDB structure: TJH for AChE (PDB ID: 4TVK), 3F9 for BChE (PDB ID: 4TPK), 3TV for hCA I (PDB ID: 4WR7), 51J for hCA II (PDB ID: 5AML), and NR4 for human maltase-glucoamylase/α-glucosidase (PDB ID: 3L4Y). The respective RMSD values between the crystallographic and redocked poses were 0.5498, 1.9056, 0.7229, 0.6997, and 1.4958 Å. Since all RMSD values were below 2.0 Å, the docking protocol was considered capable of reproducing the experimentally observed binding orientations.

## 3. Discussion

Peonidin effectively reduced the activity of the cholinergic enzymes AChE and BChE, which are involved in AD symptom reduction. Phenolic compounds exhibit a broad spectrum of pharmacological activities, including antioxidant, anti-inflammatory, antibacterial, neuroprotective, and antidiabetic effects. These biological activities may be interconnected through common mechanisms, including free-radical scavenging, metal chelation, modulation of inflammatory signaling pathways, and interactions with disease-related enzymes [27]. Plants contain phenolics with a wide variety of polarities. These substances have scavenging qualities because they contain many hydroxyl groups. Phenolic acids, tannins, and flavonoids are the primary classification categories of polyphenols that are identified by certain structural variations. These are strong antioxidants with a variety of biological properties, such as antibacterial activity [28]. One of the most prevalent types of secondary metabolites in plants is phenolic compounds. They have garnered plenty of interest as naturally occurring antioxidants due to their potent metal-chelating ability and effective free radical-scavenging qualities. Their redox properties, which allow them to donate hydrogen atoms, quench singlet oxygen, and neutralize ROS, are mainly responsible for their antioxidant capacity. A significant source of dietary antioxidants, phenolic compounds, are found in the plant kingdom. They represent a major area of study in nutrition, pharmacology, and food science due to their wide range of biological activities and exceptional antioxidant qualities [15].

Anthocyanins, a significant class of dietary phytochemicals, are plentiful in purple sweet potatoes (*Ipomoea batatas*). Previous research has shown that purple sweet potato extracts include seven main anthocyanins, which are thought to significantly contribute to their antioxidant potential. These anthocyanins include three cyanidin derivatives and four peonidin derivatives [29]. Antioxidants perform numerous functions, including hydrogen peroxide scavenging, decomposition of peroxides, elimination of reactive oxygen species, and chelation of metal ions. Reducing power, the most crucial attribute of an antioxidant, is also a result of the capacity to scavenge electrons [30].

In this study, a range of methods was employed to evaluate the antioxidant activity of peonidin, including the ABTS, DPPH, and DMPD radical scavenging assays, as well as the removal of potassium ferricyanide and cupric ions (Cu^2+^) complex [31].

In the literature on this subject, Fe^3+^ reduction absorbances 0.432 (r^2^: 0.9981) for uric acid [32], 0.739 (r^2^: 0.9478) for coumestrol [33], 0.967 (r^2^: 0.9938) for Magnofluorine [34], 1.012 (r^2^: 0.9523) for Spiraeoside [35], 1.360 (r^2^: 0.9374) for Vescalagin [36], 2.428 for tannic acid (r^2^: 0.9474) [37], 2.509 for caffeic acid phenethyl ester (r^2^: 0.9906) [38], and 2.769 for caffeic acid (r^2^: 0.9945) [39] at the same concentrations. Furthermore, as previously noted in the literature for natural phenolic substances, the absorbance values for the reduction of Cu^2+^ by Magnofluorine [34]; 0.519 (r^2^: 0.9675) for Spiraeoside [35]; 0.519 (r^2^: 0.9223) for Vescalagin [36], 0.762 (r^2^: 0.9957) for eugenol [40]; 0.780 (r^2^: 0.9981) for coumestrol [33]; 1.085 (r^2^: 0.8403) for resveratrol [41]; 0.750 (r^2^: 0.9550) for taxifolin [42]; and 1.314 (r^2^: 0.9682) for olivetol [43] at the same concentrations.

Polyphenols’ molecular structure, namely the quantity and distribution of hydroxyl groups, the existence of conjugated double bonds, and other structural characteristics, largely determine their antioxidant action. Because they can more readily donate hydrogen atoms to neutralize free radicals, polyphenols with higher hydroxyl group content often exhibit greater antioxidant activity. Additionally, the O–H bond dissociation enthalpy was reduced by conjugated double bonds and certain side-chain configurations, which promote H atom transfer and enhance radical scavenging effectiveness [21,44]. Antioxidant activity has been indicated by the unsubstituted OH groups within peonidin.

As free radicals possess unpaired electrons, they can bind to proteins, lipids, and DNA, thereby altering them. This leads to oxidative stress, which causes a range of diseases in humans. Consequently, the use of exogenous antioxidants may help to manage oxidative stress. Plants with bioactive substances like flavonoids and phenols may help reduce oxidative stress. These compounds can neutralize free radicals by providing hydrogen atoms, making them important components of antioxidants. Moreover, they exhibit beneficial structural properties for scavenging free radicals [45]. The biological, pharmacological, and nutritional significance of antioxidants is largely dependent on their capacity to scavenge free radicals and ROS. By preventing oxidative damage to cells, tissues, and food products, antioxidants improve health and product stability [46]. Because of their speed, ease of use, selectivity, affordability, and excellent repeatability, the ABTS, DPPH, and DMPD radical scavenging assays are the most widely used techniques for assessing antioxidant activity. As such, they are frequently used to evaluate the ability of pure substances to scavenge free radicals. The ABTS, DPPH, and DMPD radicals’ distinctive green-blue, purple, and pink hues, respectively, offer great analytical sensitivity and allow for precise and trustworthy spectrophotometric measurement of antioxidant activity [30]. Furthermore, peonidin can interact with the unsaturated portion of the lipid chain and function as a chain-breaking radical scavenger. The presence of H-providing substituent groups, which are essential for stability, and the delocalization capacity of the free electrons are the most advantageous structural features for the antioxidant potential of phenolics. It is well known that a phenolic molecule’s capacity to produce biological activity and operate as an antioxidant is influenced by the number and location of its -OH groups. Because of these properties, polymeric polyphenols are better antioxidants than monomeric polyphenols [47].

**Figure 4:**
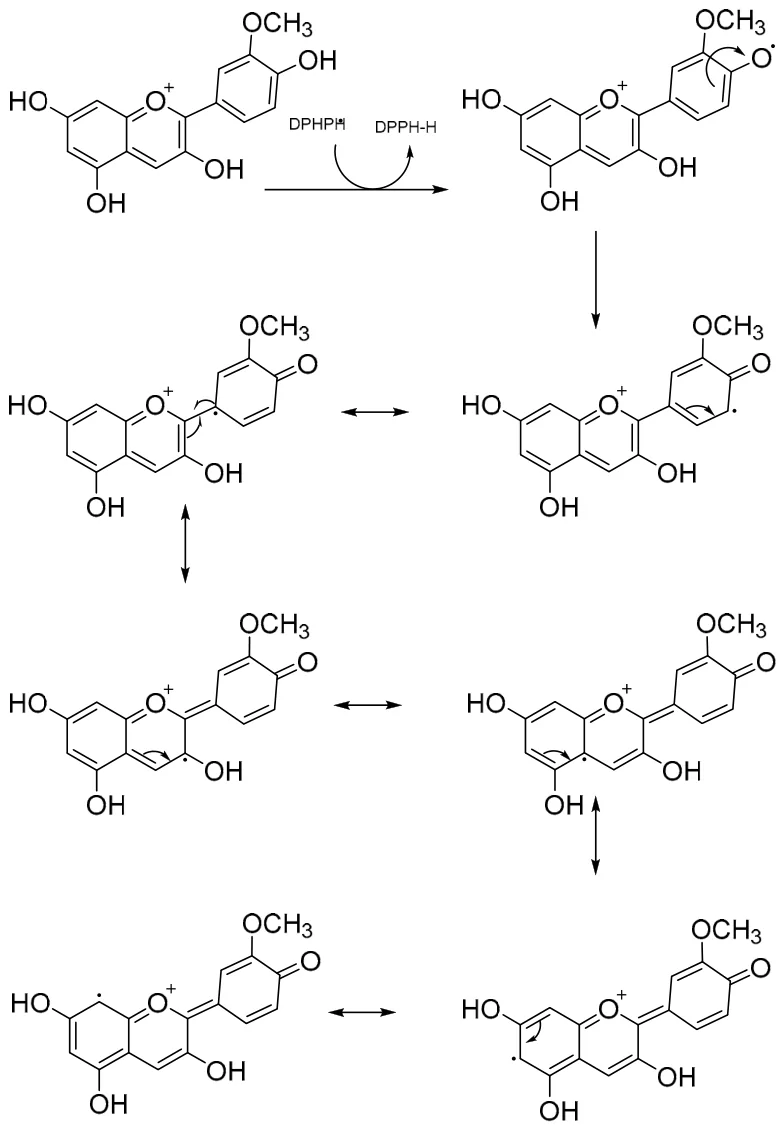
Proposed resonance stabilization mechanism for DPPH radical scavenging by peonidin.

When a hydrogen-donating antioxidant is found, the DPPH radicals are reduced and, at the end of the reaction, the non-radical DPPH-H form is created [48,49]. Many other phenolic and flavonoid antioxidants evaluated by Gulcin et al. [20] for their DPPH radical scavenging capacities are comparable to peonidin in free radical scavenging capacity. As stated in various studies, the IC_50_ value is 3.30 μg/mL for CAPE [39], 16.06 μg/mL for eugenol [40], 6.96 μg/mL for resveratrol [41], 9.65 μg/mL for Vescalagin [36], 77.00 μg/mL for taxifolin [42], 10.58 μg/mL for Magnofluorine [34], 17.77 μg/mL for olivetol [43], 20.8 μg/mL for silymarin [49], 25.95 μg/mL for coumestrol [33], 28.51 μg/mL for Spiraeoside [35], L-Adrenaline [50] at 30.6 μg/mL and curcumin [51], the first plant-derived phenolic compound, at 34.9 μg/mL. According to the study’s results, peonidin possesses the capacity to scavenge DPPH radicals. After producing DPPH-H by abstracting an electron or a hydrogen atom from peonidin, the radicals are eliminated upon contact between peonidin and DPPH (Figure 4).

According to the proposed mechanism, peonidin scavenges the DPPH radical primarily through hydrogen atom transfer from its phenolic hydroxyl groups. The electron-deficient DPPH radical abstracts a hydrogen atom from peonidin, forming stable DPPH-H and a resonance-stabilized peonidin phenoxy radical. The unpaired electron generated on peonidin is delocalized across the aromatic rings and conjugated flavylium system, which markedly reduces its reactivity. Resonance stabilization involving the oxygen-containing substituents further supports radical neutralization. Consequently, the purple DPPH radical is converted into its reduced, yellow-colored form. This mechanism demonstrates how peonidin’s hydroxyl groups and conjugated structure underpin its overall effective antioxidant and radical-scavenging activity.

The ability of organic compounds to directly combine with the ABTS radical and reduce its color was assessed using the ABTS radical scavenging test [52]. The presence of numerous hydroxyl groups attached to the aromatic ring may explain why peonidin exhibits powerful free radical scavenging activity towards DPPH and ABTS, as well as electron transfer [53]. When compared with other organic compounds, the IC_50_ value for CAPE 9.80 μg/mL [38], 7.84 μg/mL for eugenol [40], 7.48 μg/mL for Spiraeoside [35], 4.90 µg/mL for Vescalagin [36], 6.96 μg/mL for resveratrol [41], 0.83 μg/mL for taxifolin [42], 1.94 μg/mL for olivetol [43], 8.62 μg/mL for silymarin [49], L-Adrenalin at 6.93 μg/mL [50], coumestrol at 12.24 μg/mL [33], curcumin at 18.07 μg/mL [51] and Magnofluorine at 27.61 μg/mL [34].

Antioxidant molecules in test samples may readily and effectively scavenge DMPD radicals. Peonidin has been found to have higher IC_50_ values in DMPD radical elimination activities, as well as in other research investigations. For example, the IC_50_ values are 26.70 μg/mL for CAPE [39], 12.81 μg/mL for Coumestrol [33], 10.04 μg/mL for eugenol [40], 8.15 μg/mL for Spiraeoside [35], 8.73 μg/mL for Vescalagin [36], 9.5 μg/mL for resveratrol [41], 173.25 μg/mL for taxifolin [42], 19.25 μg/mL for olivetol [43], 15.6 μg/mL for L-Adrenaline [50], 15.16 μg/mL for Magnofluorine [34], and 34.5 μg/mL for curcumin [51].

Lipid peroxidation, caused by free radicals, leads to tissue damage in diabetes due to increased free radicals and the deprivation of antioxidant compounds and enzymes. Antioxidants have been shown to diminish oxidative stress, which aggravates diabetes [54]. Natural products, whether pure substances or standardized extracts, show great promise as cutting-edge medicinal interventions due to their diverse variety of chemical compositions. Plants are an affordable and abundant source for producing novel medications. Issues regarding the management of diabetes are raised by the fact that contemporary herbal medications and botanicals have more effective drug-prospective compounds with few or no adverse effects [55].

The hydrolysis metabolites of α-amylase are substrates for α-glycosidase, which generates glucose. By successfully reducing the rate of starch hydrolysis, inhibiting the activity of these enzymes can assist in managing postprandial hyperglycemia. Restricting the digestion and absorption of carbohydrates is the goal of several diabetic drugs. Miglitol, Voglibose, and acarbose are a few enzyme inhibitors that are often used in therapy. However, adverse side effects such as diarrhea, gas, meteorism, and bloating in the abdomen frequently accompany these medications’ efficacy. Because of these major side effects, experts are seeking alternative therapies with minimal to no negative consequences [56]. An elevated level of starch hydrolysis by pancreatic α-amylase and a higher level of absorption of glucose by intestinal α-glycosidase can lead to hyperglycemia, or an abrupt rise in blood sugar, in type II diabetes. Thus, moderate inhibition of pancreatic α-amylase and substantial inhibition of α-glycosidase are effective methods for controlling type II diabetes. Because of their interactions with the transmembrane protein GLUT1 and the pancreatic enzymes α-amylase and β-glycosidase, anthocyanins produced from *Chakhao poreiton* have high prebiotic and antidiabetic characteristics, as well as contributions to their antioxidant activity [57]. The regulation of blood sugar levels by inhibiting the α-glycosidase enzyme is recognized to be one of the most essential approaches to avoid the clinical complications in T2DM that impact the heart, kidneys, eyes, neurological systems, and other bodily systems. T2DM frequently results in several illnesses, including heart disease, stroke, renal failure, erectile dysfunction, blindness, visual impairment, lower extremity amputations, and inadequate wound healing [58]. Peonidin had an effective IC_50_ and Ki values of 33.01 and 15.14 ± 4.93 nM based on the results of α-glycosidase inhibition, which was comparable to the starch inhibitor medication, IC_50_ value of Acarbose (22,800 µM). The onset, course, and consequences of diabetes mellitus are significantly and often influenced by elevated oxidative stress.

Currently authorized drugs for AD include cholinesterase inhibitors (ChEIs), such as memantine, galantamine, rivastigmine, and donepezil. Nevertheless, these drugs cannot halt or alter the course of AD; they can only momentarily lessen symptoms. Their clinical use is limited due to significant side effects and poor compliance [59]. High anthocyanin concentration (585 mg cyanidin-3-O-glucoside and 24 mg peonidin-3-O-glucoside per gram of the rich extract) is found in the anthocyanin-rich part of black rice (*Oryza sativa*) bran. It has been discovered that this drug lessens neurodegeneration [60]. The cholinergic hypothesis, which contends that a shortage of cholinergic activity in the brain is the reason for patients’ memory loss, is the foundation of several therapeutic approaches. Using AChE inhibitors to raise ACh levels in the synaptic cleft is one of the most promising methods for treating this illness [61].

In conclusion, when isolated from their primary sources using environmentally friendly extraction methods, plant-derived natural secondary compounds can be a significant source of innovative drugs with very few adverse effects. The active polyphenolic peonidin molecule may have broad pharmaceutical effects on AD. This research recorded the IC_50_ values of peonidin on cholinergic enzymes. Furthermore, peonidin inhibited AChE and BChE with K_i_ values of 2.99 ± 0.23 and 9.76 ± 3.23 nM, respectively. Tacrine, used as the reference inhibitor, exhibited corresponding K_i_ values of 1.85 ± 0.31 and 1.52 ± 0.01 nM. Table 3 shows that tacrine has a lower Ki value than peonidin on AChE and BChE enzymes. Moreover, when examining Ki levels for AChE and inhibition in the previous literature, many organic compounds have been recorded as follows: 0.518 nM for CAPE [39], 3.4 nM for Spiraeoside [35], 5.13 nM for olivetol [43], 5.87 nM for Vescalagin [36], 10.25 nM for Magnofluorine [34],16.70 nM for taxifolin [40], and 21.43 nM for coumestrol [33]. There are no current studies on peonidin with pharmacological activity; this is the first study reporting its activity as an inhibitor of AChE and BChE. Furthermore, experimental observations show that this chemical compound effectively inhibits ROS and free radical aggregation.

Phenolic and polyphenolic compounds are classified as CAIs because they show a wide range of biological effects. Several physiologically relevant isoforms are potently inhibited by many of these compounds. To discover new CA isoenzyme inhibitors, aromatic compounds can be intriguing precursor molecules [43]. Phenolics lose protons (H^+^) from their hydroxyl groups and change their pH from slightly acidic to highly water-soluble phenolate anions. Moreover, phenols have advantageous inhibitory effects on CA isoenzymes [62]. The natural anthocyanins delphinidin, luteolinidin, and peonidin are non-toxic to human retinal pigment epithelium and retinal ganglion cells; luteolinidin and peonidin increased the survival rates of retinal ganglion cells after exposure to H_2_O_2_ [63]. The strongest aspect of this study is the detailed investigation of the inhibitory effects of peonidin towards CA I and CA II enzymes, demonstrating that the results are efficiently compared to the acetazolamide standard. Peonidin showed non-competitive inhibition against all enzymes in this research (Figure 3). Since peonidin has not been previously investigated in relation to inhibition of hCA I, hCA II, AChE, α-glycosidase and BChE enzymes, further studies are needed. This thorough and important investigation of peonidin in antioxidant assays and metabolic enzyme inhibition is the first of its type conducted by our team. Peonidin compounds have been the subject of extremely few and inadequate enzyme investigations. The findings of this investigation can be used as a model for future research. Pharmacology and medicine will benefit from the implementation of the study’s findings.

Peonidin is a flavonoid belonging to the anthocyanidin class that, thanks to its phenolic hydroxyl groups, can interact with the lipid bilayer of the bacterial cell membrane and disrupt membrane permeability [64]. In Gram-positive bacteria, the porous structure of the peptidoglycan layer allows peonidin to reach the cell membrane easily; this is consistent with the significant inhibition observed at low concentrations in *S. aureus* [64]. In Gram-negative bacteria, the outer membrane forms a significant barrier limiting flavonoid passage. However, the higher inhibition observed in *E. coli* suggests that peonidin may be able to partially penetrate the outer membrane or enter the cell via porin channels [64,65]. The lack of efficacy observed in *P. aeruginosa* and *K. pneumoniae* may be attributed to intrinsic resistance mechanisms such as the bacteria’s low-permeability outer membranes, strong efflux pump systems, and capsule structures [66]. The literature reports that the antibacterial effects of flavonoids, particularly in Gram-positive bacteria, primarily *S. aureus*, are observed with low MIC values and that this efficacy is largely based on a cell membrane-targeted mechanism [66]. Furthermore, a study using peonidin-based anthocyanins reported MIC values of 0.25–0.50 mg/mL for *S. aureus* and *Salmonella typhimurium* [67]. These findings demonstrate that the results obtained in our study are consistent with the literature.

Molecular docking analyses demonstrated that peonidin exhibits target-dependent binding affinity toward the investigated enzymes, with the strongest interactions observed for AChE, α-glycosidase, and BChE, whereas comparatively weaker binding was predicted for CA isoforms I and II (Table 4). Although the reference inhibitors generally exhibited more favorable docking scores, an exception was observed for BChE, for which peonidin showed a more favorable docking score than tacrine (−6.868 and −5.518, respectively). Nevertheless, tacrine displayed substantially stronger experimental BChE inhibition than peonidin, with K_i_ values of 1.52 ± 0.01 and 9.76 ± 3.23 nM, respectively. This discrepancy indicates that docking scores alone do not directly predict inhibitory potency. Factors not fully represented by the docking model—including protein flexibility, ligand solvation and desolvation, entropic contributions, protonation states, binding kinetics, and assay conditions—may influence the experimentally observed activity. Therefore, the docking results should be interpreted as qualitative evidence of plausible binding orientations and interactions rather than as a quantitative measure of inhibitory potency.

The present findings are based primarily on in vitro spectrophotometric biochemical assays. Although multiple complementary antioxidant methods, enzyme kinetic analyses, appropriate blanks, and molecular docking were employed, these approaches do not substitute for orthogonal binding measurements, cell-based functional assays, or in vivo validation. Future studies should confirm the observed activities using techniques such as isothermal titration calorimetry or surface plasmon resonance, cellular oxidative-stress and enzyme-activity models, and appropriately designed in vivo experiments. Therefore, the current results should be interpreted as preliminary biochemical evidence.

## 4. Materials and Methods

### 4.1. Chemicals and Materials

The chemicals peonidin, BHA, BHT, Trolox, α-tocopherol, DPPH, ABTS, and DMPD, butyrylthiocholine iodide, acetylthiocholine iodide, and peonidin chloride were purchased from Sigma (Sigma-Aldrich GmbH, Sternheim, Germany). *Staphylococcus aureus* ATCC 25923, *Escherichia coli* ATCC 8739, *Pseudomonas aeruginosa* 27853, and *Klebsiella pneumoniae* ATCC 13833 were bought from the American Type Culture Collection (ATCC, Manassas, VA, USA).

### 4.2. Reducing Capacity Experiments

As in a previous study [68], the reducing capacity of peonidin towards Fe^3+^ was determined. To determine the reducing effect of peonidin on Fe^3+^, the absorbances of peonidin and the standards were recorded at 700 nm [69]. To measure the reducing capacity of peonidin towards Cu^2+^, the method described by Apak et al. was used [70]. A spectrophotometer was used to capture the absorbance values at 450 nm following the completion of the required experimental procedures. The foundation of the FRAP reduction power is the reduction of Fe^3+^-TPTZ in an acidic medium [15]. The reduced form of Fe^2+^-TPTZ was detected at 593 nm spectrophotometrically.

### 4.3. Radical Scavenging Activities

The Blois method [71] was used to measure the DPPH scavenging activity of Peonidin. The DPPH solution was prepared one day before the measurement. The mixture tube was wrapped in aluminum foil to seal it and was kept in the dark at 4 °C for 16 h. After preparing the 0.1 mM DPPH mixture in ethanol, 0.5 mL of this solution was added to 2 mL of peonidin sample solution containing varying amounts (10–30 µg/mL) of ethanol. After vortexing the peonidin samples, they were kept in the dark at 30 °C for 30 min. Following comparison with a blank sample, the absorbances were recorded at 517 nm [39]. The DPPH∙ absorbance measured at 517 nm was calculated as given previously [72]. The ABTS^•+^ scavenging activity of peonidin was determined in agreement with a previously conducted study and measured at 734 nm [73]. ABTS^•+^ was prepared by combining 2 mM ABTS in water with 2.45 mM potassium persulphate (K_2_S_2_O_8_), and the solution was kept at room temperature in the dark for 6 h. Although ABTS oxidized quickly, it took over six hours for its absorbance to stabilize and peak. When stored in darkness at room temperature, the radical cation stays stable in this condition for almost two days. To set up the experiment, the mixture was diluted in phosphate buffer (pH 7.4) in a 1 mL cuvette to attain an absorbance of 0.800 ± 0.05 at 734 nm, then equilibrated at the test temperature of 30 °C. Subsequently, 3 mL samples of peonidin in ethanol at different combinations (10–30 µg/mL) were associated with 1 mL of ABTS^•+^ solution. After stirring for 30 min, absorbances were measured, and the radical scavenging percentages were adjusted for each amount by comparing them with a control sample lacking a scavenger. The rate of decrease in absorbance was used to determine the extent of color loss [74]. Peonidin was evaluated for its DMPD^•+^ reduction capacity using the Fogliano method [75], as previously described. For sample measurement, 750 µL of DMPD^•+^ (0.1 M) was supplemented to 1 mL of acetate buffer (pH 5.3, 0.1 M) containing peonidin (10–30 µg/mL). Subsequently, 0.25 mL of 50 mM FeCl_3_ was added to the solution. Finally, the absorbances of DMPD^•+^ were measured spectrophotometrically at 505 nm [35].

### 4.4. Anti-Alzheimer’s Disease Experiments

Likewise, Ellman’s method was used to assess the inhibitory outcomes of peonidin on AChE/BChE using serum from the electric eel (*Electrophorus electricus*) [76]. The reaction was carried out using AChE serum obtained from the electric eel. In short, the enzyme solution (50 μL, 5.32 × 10^−3^ EU) in buffer (1.0 M Tris/HCl, 100 μL, pH 8.0) was mixed with a certain quantity of peonidin (10–30 µg/mL). The solutions were left to stand at 20 °C for 10 min. Then, 50 μL of mixtures containing DTNB (5,5′-dithio-bis(2-nitrobenzoic acid)) (0.5 mM) and acetylcholine iodide (AChI) were added. As a result, the reaction commenced, and the absorbances of the solutions were measured at 412 nm using a spectrophotometer [77].

### 4.5. Antidiabetic Experiment

Complying with the protocol described by Tao et al. [24], the inhibitory effect of peonidin on α-glycosidase was evaluated using p-nitrophenyl-D-glucopyranoside (p-NPG) as the substrate. The absorbances of the samples were recorded at 405 nm. The quantity of enzyme catalyzing 1.0 mol of substrate per minute (at pH 7.4) was taken as one α-glycosidase unit.

### 4.6. Antiglaucoma Experiments

Residual erythrocyte fractions from human blood specimens that had completed routine diagnostic testing in the hospital biochemistry laboratory were used as the source of hCA II. These specimens would otherwise have been discarded as laboratory waste and were irreversibly anonymized before transfer to the researchers. No samples were collected specifically for this study, and the researchers had no access to donor identities, clinical records, or demographic information. The erythrocytes were centrifuged at 10,000× *g* for 30 min. After separating the serum, solid Tris was used to raise the pH to 8.7 [20,78]. Sepharose-4B-L-Tyrosine sulphanilamide affinity column chromatography was employed to isolate the CA II isoenzyme [79]. Samples were loaded onto the affinity column and equilibrated using Tris-Na_2_SO_4_/HCl (22 mM/25 mM, pH 8.7). Sodium acetate/NaClO_4_ (0.5 M, pH 5.6, 25 °C) was subsequently used to elute the hCA II isoenzyme. The Bradford method [80] was employed to calculate protein concentration during the purification process. Bovine serum albumin was used as a reference protein. In accordance with preceding studies, the purity of the hCA II isoform was determined by SDS-PAGE [81]. Esterase activity was measured during the isolation and inhibition processes of hCA II. Absorbance changes at 348 nm were measured to assess CA II isoenzyme activity [82].

### 4.7. Inhibition Parameters

Graphs plotting activity (%) against peonidin were applied to calculate the IC_50_ value. The proportion of enzyme inhibition in response to rising peonidin quantities was determined spectrophotometrically to get the IC_50_ values. From this data, graphs were subsequently created. Finally, the IC_50_ and r^2^ values were calculated from mathematical formulae generated from the graph [34]. Lineweaver-Burk plots were used to compute Ki and other parameters. The Ki values were determined using these graphical outcomes [83].

### 4.8. Molecular Docking Study

The inhibitory effects of the peonidin molecule on CA I, CA II, AChE, BChE, and α-glycosidase enzymes were investigated experimentally. In silico, the interactions of the peonidin molecule with CA I, CA II, AChE, BChE, and α-glycosidase enzymes were identified using the molecular docking approach. The Schrödinger Molecular Modeling Suite Maestro 13.5 software (https://doi.org/10.1002/slct.202303054) was used to conduct the investigations. In the initial phase of the molecular docking studies, the molecular structures of peonidin and the clinical inhibitors of CA I, II, AChE, BChE, and α-glycosidase enzymes, acetazolamide, tacrine, and acarbose, were constructed using SMILES codes obtained from the PubChem online database. Optimization studies were performed using Maestro’s LigPrep software (Table 7) (Schrödinger Inc. Version 2023-1: LigPrep, LLC, New York, NY, USA, 2023). The enzyme structures were obtained from the RCSB Protein Data Bank website (PDB identifiers: CA I: 4WR7, CA II: 5AML, AChE: 4TVK, BChE: 4TPK, α-glycosidase: 3L4Y). The Schrödinger program version 13.5.128’s protein production wizard tool was used to conduct protein optimization research. Receptor grids were prepared for ligand docking analysis as part of the ongoing research. Receptor grid preparation makes it possible to identify the location and dimensions of the protein’s active site. The Schrödinger Suite 2022-3’s receptor grid creation tool in Maestro 13.5 was used for these investigations. The ligand docking program in the Schrödinger Molecular Modelling Suite (Maestro Version 13.5) was used to ascertain the compounds’ possible interactions and binding affinities with each enzyme.

### 4.9. Preparation of Microorganisms and Determination of Antimicrobial Activity

The disk diffusion test was used to assess the antibacterial activity of peonidin. The bacterial agar cultures used in the experiment were prepared as described by Gulcin et al. [84]. Blood agar was used to culture microorganisms. Tryptone soy broth was infected with the microorganisms employed in the experiment. Tryptone soy broth, a very nutritious, adaptable medium suggested for general laboratory usage, was used to cultivate facultative anaerobes and aerobes, including certain fungi. For a whole day, the cultures were incubated at 37 °C. For the antimicrobial test, 50 μL of peonidin samples were placed on sterile filter paper disks with a diameter of 6 mm. The diffusion technique outlined in the Clinical and Laboratory Standards Institute [85] recommendations was then used to evaluate susceptibility on Mueller-Hinton agar.

The peonidin molecule was tested against Gram-positive and Gram-negative bacteria to measure its antimicrobial activity. *S. aureus* was selected as the Gram-positive bacterium, and *P. aeruginosa*, *K. pneumoniae*, and *E. coli* were selected as the Gram-negative bacteria. The bacterial cells were cultured on nutrient agar at 37 °C for 18–24 h. Following cultivation, the cells were transferred to 0.9% NaCl and diluted in the test agar medium until an OD600 nm value of 0.5 McFarland (1.3 × 10^8^ CFU/mL) was achieved. A McFarland standard curve was prepared using mixtures of varying turbidity, prepared with 1% BaCl_2_ and 1% H_2_SO_4_ solutions, to cover the range of 0.5–4.0 (0.5–1.0–2.0–3.0–4.0) McFarland units. Samples of different concentrations were loaded onto blank disks. DMSO and tetracycline-loaded disks were utilized as negative and positive controls. The sample-loaded dried disks were transferred to the test medium and incubated statically at 37 °C for 24 h. Following incubation, the diameters of the inhibition zones in the Petri dish were recorded using a ruler [86,87].

In the antimicrobial test, results were determined by measuring the zones of growth inhibition around disks containing antibiotics and peonidin. The disk’s surrounding clear zones of inhibition showed antimicrobial activity. The antimicrobial test results were evaluated in accordance with the guidelines proposed by the Clinical and Laboratory Standards Institute (CLSI, 2019) [88].

#### Statistical Analysis

Each measurement was repeated three times. The findings are displayed as mean ± SD. The Independent *t*-test was used to determine if the mean value differences were statistically significant. A statistically significant *p*-value was defined as less than 0.05.

## 5. Conclusions

Anthocyanins are natural compounds found in colorful plants such as blueberries, cherries, and grapes, and are associated with a reduced risk of chronic diseases, T2DM, and neurodegenerative disorders. They support health by regulating oxidative stress, providing defense against microorganisms, and regulating glucose metabolism. In this study, peonidin, an anthocyanin compound, exhibits antioxidant activity on the molecular level by inhibiting crucial metabolic enzymes. The expression of genetic material through modifications in the activity of enzymes and proteins results in the regulation of hormonal and metabolic cycles during the organism’s healthy life cycle. Numerous common, degenerative, progressive aging, chronic, shortening lifespans, and deadly illnesses can be avoided, stopped, or lessened when the activities of enzymes involved in the regulatory processes are blocked, decreased, or managed. Because of its hydroxyl groups and the molecules, it interacts with in vitro, peonidin, a flavonoid chemical, has demonstrated antioxidant, anti-AD, antidiabetic, antiglaucoma, and antiepileptic properties. It may also exhibit these effects in vivo. This study, regarded as the most thorough molecular-based investigation to date, offers a thorough scientific and quantitative characterization of peonidin, which has been mentioned before in the literature mostly through qualitative studies. It has been found through experimentation that, when measured against synthetic antioxidants, the peonidin molecule can eliminate free radicals and ROS using a variety of analytical techniques.

This research demonstrates that peonidin, a reliable plant-based flavonoid antioxidant, may be employed in pharmaceutical research and the food sector to postpone or stop oxidative degradation. Peonidin, for instance, exhibited an IC_50_ value of 41.63 μg/mL for DPPH^•^ radical scavenging activity. In contrast, BHA, BHT, Trolox, and α-Tocopherol had IC_50_ values of 8.45, 23.10, 6.30, and 18.73 μg/mL, respectively, for the same activity. Peonidin’s antioxidant function may protect the values of nutrients and extend the lifespan of medications and dietary products. The study’s findings demonstrated that peonidin efficiently inhibits CA I, II, BChE, AChE, and α-glycosidase. According to the research, peonidin’s Ki values against hCA I, hCA II, AChE, BChE, and α-glycosidase are 114.33, 63.02, 2.99, 9.76, and 15.14 nM, respectively. Collectively, the molecular docking findings revealed that the peonidin compound can effectively interact with the catalytic regions of the target enzymes, with the strongest predicted interactions observed for cholinesterases and α-glycosidase, providing molecular insights into its inhibitory potential and biological activity. Some prevalent chronic disorders, including AD, glaucoma, and diabetes mellitus, have been linked to these enzymes, whose inhibition has been examined. Future research should include experiments usingin vitro cell cultures and in vivo experimental animals to ascertain the impact of the peonidin molecule on target disease pathways.

## Figures and Tables

**Table 1:** The Fe^3+^, Cu^2+^, and Fe^3+^-TPTZ reducing capacity of peonidin and the standard antioxidants at a concentration of 30 μg/mL.

Antioxidants	Fe^3+^ Reducing		Cu^2+^ Reducing		Fe^3+^-TPTZ Reducing	
	λ_700_	r^2^	λ_450_	r^2^	λ_593_	r^2^
BHA	1.646 ± 0.027	0.7418	1.783 ± 0.045	0.6636	1.322 ± 0.078	0.9842
BHT	0.440 ± 0.006	0.7230	2.128 ± 0.008	0.7744	2.065 ± 0.077	0.8647
Trolox	1.896 ± 0.141	0.9086	1.635 ± 0.015	0.7407	2.089 ± 0.012	0.8013
α-Tocopherol	1.297 ± 0.014	0.9896	1.815 ± 0.058	0.9906	2.256 ± 0.112	0.9527
Peonidin	0.637 ± 0.020	0.9970	0.701 ± 0.020	0.9992	1.072 ± 0.020	0.9347

**Table 2:** IC_50_ (μg/mL) values for the DPPH^•^, ABTS^•+^ and DMPD^•+^ scavenging activities of peonidin and reference antioxidants.

Antioxidants	DPPH^•^ Scavenging		ABTS^•+^ Scavenging		DMPD^•+^ Scavenging	
	IC_50_	r^2^	IC_50_	r^2^	IC_50_	r^2^
BHA	8.45	0.9952	12.82	0.9966	40.77	0.9872
BHT	23.10	0.9818	11.78	0.9468	-	-
Trolox	6.30	0.9239	12.67	0.9899	10.19	0.6735
α-Tocopherol	18.73	0.9966	10.83	0.9304	-	-
Peonidin	41.63	0.9642	15.40	0.9908	31.53	0.6918

**Table 4:** Enzyme docking and binding energy scores for CA I, II, AChE, BChE, and α-glycosidase compounds.

Enzymes	Compounds	Docking Score	XP Gscore	Glide Gscore	Glide Emodel
CA I (PDB: 4WR7)	Peonidin	−4.369	−4.490	−4.490	−42.441
	Acetazolamide	−6.038	−6.944	−6.944	−50.025
CA II (PDB: 5AML)	Peonidin	−2.330	−2.451	−2.451	−41.905
	Acetazolamide	−4.180	−4.546	−4.546	−49.970
AChE (PDB: 4TVK)	Peonidin	−8.876	−8.997	−8.997	−42.919
	Tacrine	−13.753	−13.753	−13.753	−61.522
BChE (PDB: 4TPK)	Peonidin	−6.868	−6.988	−6.988	−59.369
	Tacrine	−5.518	−5.519	−5.519	−42.567
α-Glycosidase (PDB: 3L4Y)	Peonidin	−8.058	−8.179	−8.179	−60.408
	Acarbose	−15.395	−15.728	−15.728	−77.262

**Table 5:** Molecular docking studies of the compounds found in the extract with enzymes: a summary of the findings.

S. No	Ligand	Pocket Grid Dimension of Protein	Residues Involved in Hydrophobic Interactions and Distances (Å)
**1**	Peonidin-CA I	Grid Box Center coordinates center_*x* = 11.685 center_*y* = 36.093 center_*z* = 17.068 Grid Box Dimension (Å) size_*x* = 10 size_*y* = 10 size_*z* = 10	HIS 94 (5.02)-π–π stacking
**2**	Acetazolamide-CA I		HIS 199 (2.08), HIS 200 (2.52)-H Bond, HIS 94 (5.44)-π–π stacking, ZN 301 (2.11)-salt bridge
**3**	Peonidin-CA II	Grid Box Center coordinates center_*x* = −4.171 center_*y* = 4.270 center_*z* = 14.458 Grid Box Dimension (Å) size_*x* = 10 size_*y* = 10 size_*z* = 10	ASN 67 (2.09), THR 199 (2.22)-H Bond
**4**	Acetazolamide-CA II		GLN 92 (1.99), HIE 119 (2.27), THR 199 (2.03)-H Bond, HIS 94 (5.27) π–π stacking
**5**	Peonidin-AChE	Grid Box Center coordinates center_*x* = −52.437 center_*y* = 30.244 center_*z* = −43.435 Grid Box Dimension (Å) size_*x* = 10 size_*y* = 10 size_*z* = 10	PHE 330 (4.52), TYR 121(5.04)-π–π stacking, PHE 288 (2.15)-H Bond
**6**	Tacrine-AChE		PHE 330 (3.62, 3.96), TRP84 (3.62, 4.00)-π–π stacking, HIS440 (1.92) H bond, TRP 84 (4.15, 4.20), PHE330 (4.05) pi cation
**7**	Peonidin-BChE	Grid Box Center coordinates center_*x* = 4.424 center_*y* = 10.528 center_*z* = 13.925 Grid Box Dimension (Å) size_*x* = 10 size_*y* = 10 size_*z* = 10	TRP 82 (5.45), PHE 329 (5.10)-π–π stacking; LEU 286 (2.03), ALA 328 (2.48)-H bond
**8**	Tacrine-BChE		TRP 82 (3.61, 4.61)-π–π stacking; HIS 438 (2.02)-H bond
**9**	Peonidine-Glycosidase	Grid Box Center coordinates center_*x* = −1.961 center_*y* = −19.186 center_*z* = −21.048 Grid Box Dimension (Å) size_*x* = 10 size_*y* = 10 size_*z* = 10	TRP 406 (5.21)-π–π stacking; ASP 327 (1.99), ASP 542 (2.47)-H bond
**10**	Acarbose-Glycosidase		ASP 542 (1.84), ARG526 (1.83, 1.91), ASP203 (1.65), HIS 600 (2.26), ASH 327 (1.89), LYS 480 (2.36)-H bond ASP 443 (4.72)–salt bridge distance

**Table 6:** 2D and 3D zoomed-in view placement images.

Docking	2D-View	3D-Zoomed View
Peonidin-CA I	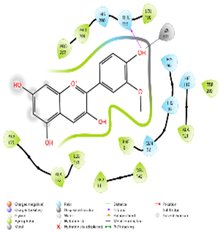	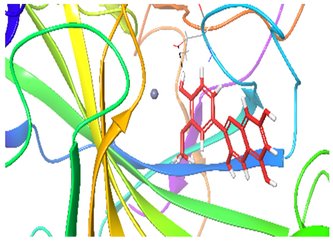
Acetazolamide-CA I	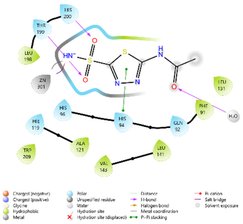	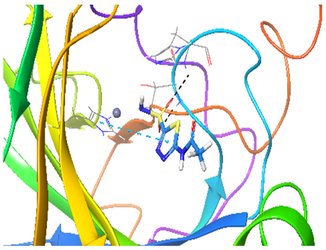
Peonidin-CA II	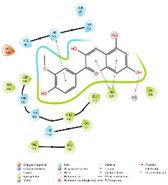	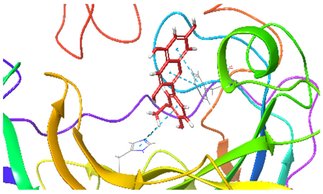
Acetazolamide-CA II	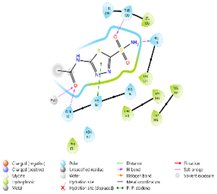	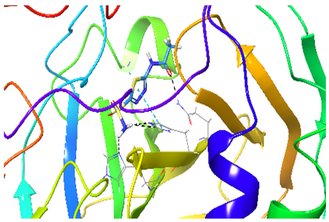
Peonidin-AChE	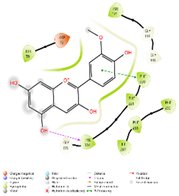	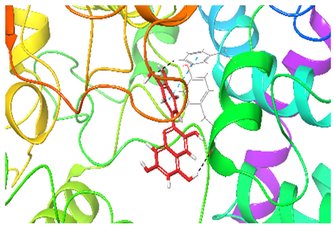
Tacrine-AChE	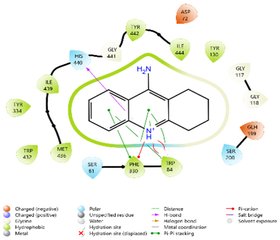	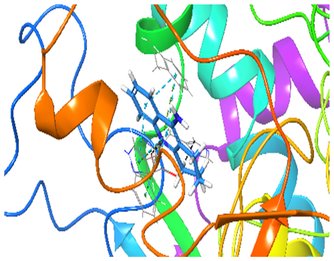
Peonidin-BChE	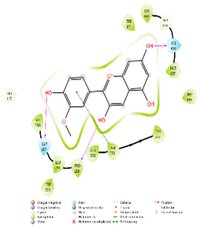	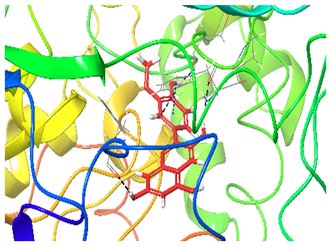
Tacrine-BChE	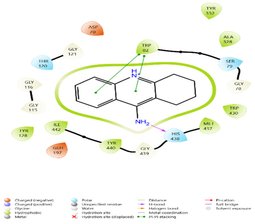	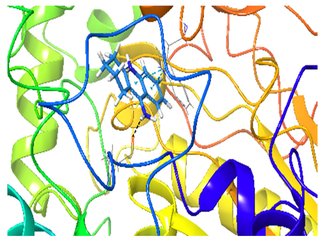
Peonidin-α-Glycosidase	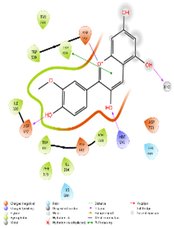	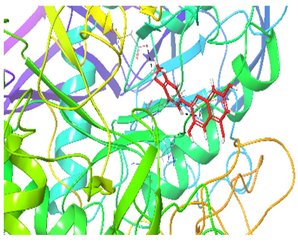
Acarbose-α-Glycosidase	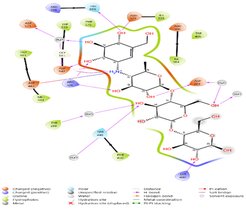	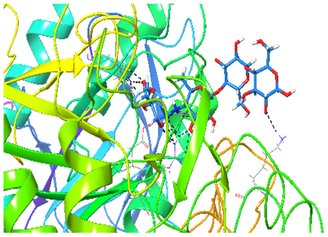

**Table 7:** PubChem CID codes for ligands used in molecular docking studies.

Ligand	PubChem CID Code	References
Peonidin	CID164544	https://pubchem.ncbi.nlm.nih.gov/#query=peonidin (accessed on 3 June 2026)
Acetazolamide	CID1986	https://pubchem.ncbi.nlm.nih.gov/compound/1986 (accessed on 3 June 2026)
Tacrine	CID1935	https://pubchem.ncbi.nlm.nih.gov/compound/1935 (accessed on 3 June 2026)
Acarbose	CID41774	https://pubchem.ncbi.nlm.nih.gov/compound/41774 (accessed on 3 June 2026)

## Data Availability

The original contributions presented in this study are included in the article. Further inquiries can be directed to the corresponding authors.
